# Obliteration of the Lachrymal Passages as a Means of Relief from Stricture

**Published:** 1881-04

**Authors:** Chas. W. Miles

**Affiliations:** Jordan Station, Ky.


### OBLITERATION OF THE LACHRYMAL PASSAGES AS A MEANS OF RELIEF FROM STRICTURE.

CHAS. W. MILES, M.D., JORDAN STATION, KY.

Read before the Southwestern Kentucky Medical Association, Nov., 1880.

It has occurred to me on two occasions to obliterate the nasal duct for the relief of stricture, and I have thought that it might not prove uninteresting to those of you who practice surgery to recount some of the advantages to be gained by this procedure in certain cases and under certain circumstances.

The more I see of the operation the more am I possessed with the conviction that in a country practice it is, in a great many cases, the most desirable, and at the same time the most certain of all methods of cure.

It often occurs to the surgeon that a patient from a distance will present himself demanding immediate relief, on account of an inability to remain long from his business. Again, every surgeon is aware of the long, tedious and painful course of treatment necessary to effect a cure in a great many cases by dilatation.

It was under such circumstances as these that I was first induced to resort to the operation. The patient, a gentleman from Tipton County, Tennessee, had had a very obstinate stricture of several years' standing. The operation was so exceedingly satisfactory that I have since been tempted to repeat it.

The procedure which I adopted differed in no essential particular from the ordinary method as laid down in the text-books, and is very easy and simple of performance.

An incision extending to the upper extremity of the sac is required to secure a good result.

Almost every surgeon has his favorite caustic with which to effect destruction of the sac. Among others, nitrate of silver has quite an extended reputation.

Were it in reach of all, I think the galvano-cautery would supersede all others. As it is not, I much prefer a hot iron, of proper shape, to all others, as being most readily obtained, easily managed and most certain in its results.

The great objection to the operation, stillicidium, is, I think, in a vast majority of cases, wholly without foundation.

I have under observation at this time a patient of mine, whom I carried to Dr. Noyes, of New York, some years ago, for the relief of a bony obstruction, and, notwithstanding the fact that there was an excessive secretion of tears for years prior to the operation, sufficient, indeed, to keep the lid and the integument beneath continually irritated, yet at no time since his recovery from the immediate effects of the operation has there been the least trouble from an overflow of tears.

Nature, according to my observation, seems to accommodate herself to this new state of affairs to a degree that is quite surprising.

In one case which passed under my observation in the service of Dr. Noyes, at the New York Eye and Ear Infirmary, there was a secondary hemorrhage on the fourth or fifth day caused by an intense malarial fever.

After my very limited experience I must candidly confess, and that too in the face of the fact that it is rarely recommended by the authorities; that were I called upon to treat a stricture of the nasal duct that was at all obstinate under the catheter, unless forbidden for cosmetic reasons, I should undoubtedly resort to obliteration.—*Medical Herald.*

